# Cutaneous Leishmaniasis Caused by *Leishmania infantum*, Israel, 2018–2021

**DOI:** 10.3201/eid2905.221812

**Published:** 2023-05

**Authors:** Michal Solomon, Nadav Astman, Karin Warshavsky, Aviv Barzilai, Tal Meningher, Dror Avni, Eli Schwartz

**Affiliations:** Chaim Sheba Medical Center, Tel Hashomer, Israel (M. Solomon, N. Astman, K. Warshavsky, A. Barzilai, T. Meningher, D. Avni, E. Schwartz);; Tel Aviv University, Tel Aviv, Israel (M. Solomon, N. Astman, K. Warshavsky, A. Barzilai, D. Avni, E. Schwartz)

**Keywords:** cutaneous leishmaniasis, *Leishmania infantum*, parasites, zoonoses, emerging infection, Israel

## Abstract

Cutaneous leishmaniasis (CL) is endemic to Israel. Previously, CL caused by *Leishmania infantum* had been reported in Israel only once (in 2016). We report 8 *L. infantum* CL cases; 7 occurred during 2020–2021. None of the patients had systemic disease. *L. infantum* CL may be an emerging infection in Israel.

*Leishmania* is a protozoan that causes a wide variety of diseases. Clinical manifestations include cutaneous leishmaniasis (CL), visceral leishmaniasis (VL), or mucocutaneous leishmaniasis, depending mainly on the specific causative leishmania species (*1*). Old World CL is classically attributable to *L. major* and *L. tropica*. However, *L. infantum*, which usually causes VL, recently has been considered as a causative agent of CL (*2*).

CL is endemic to Israel and has been attributed almost exclusively to infection with *L. major* and, more recently, to *L. tropica* (*3*). However, human VL caused by an *L. donovani* substrain, *L. infantum*, has been reported, albeit rarely, in Israel (*4*). The reservoir is dogs, and the epidemiology of the disease among dogs indicates wide distribution in Israel (*5*). Nevertheless, cases of CL caused by *L. infantum* were previously reported in the Middle East and worldwide. In Israel, CL caused by *L. infantum* was reported in 2016 (*6*); that infection was acquired in the southern part of Israel, known to be endemic for *L. major.* In this article, we report 8 cases of *L. infantum* CL observed in Israel during 2018–2021.

## The Study

All patients were seen at Chaim Sheba Medical Center (Tel Hashomer, Israel). We defined *L. infantum* CL as cutaneous lesions (ulcers, nodules, or papules) clinically compatible with leishmaniasis and a PCR result positive for *L. infantum.* The study was approved by the Chaim Sheba Medical Center Institutional Review Board (protocol approval no. 7274–09).

We took tissue specimens from suspected skin lesions and extracted DNA from dried blood spots by using the QIAamp DNA Mini Kit (QIAGEN, https://www.qiagen.com). We analyzed the samples for internal transcribed spacer 1 PCR by using 10 μM primers (LITSR 5′-CTGGATCATTTTCCGATG-3′ and L5.8S 5′-TGATACCACTTATCGCACTT-3′) (*7*). We analyzed the amplicons on 4% agarose gel. We sent the *L. infantum*–positive samples to Hylabs (Rehovot, Israel) for a second validation and sequencing.

During 2016–2021, our laboratory at Chaim Sheba Medical Center diagnosed 609 cases of leishmania (Table 1). Among those, 8 cases (1.3%) were attributable to *L. infantum.* All of the cases were confirmed by PCR and further sequencing.

**Table 1:** Leishmaniasis cases diagnosed (N = 609), by *Leishmania* species, at the laboratory at Chaim Sheba Medical Center, Tel Hashomer, Israel, 2016–2021*

*Leishmania* species	No. (%) cases
*L. major*	403 (66.1)
*L. tropica*	175 (28.7)
*L. braziliensis*	23 (3.7)
*L. infantum*	8 (1.3)

**Figure 1:**
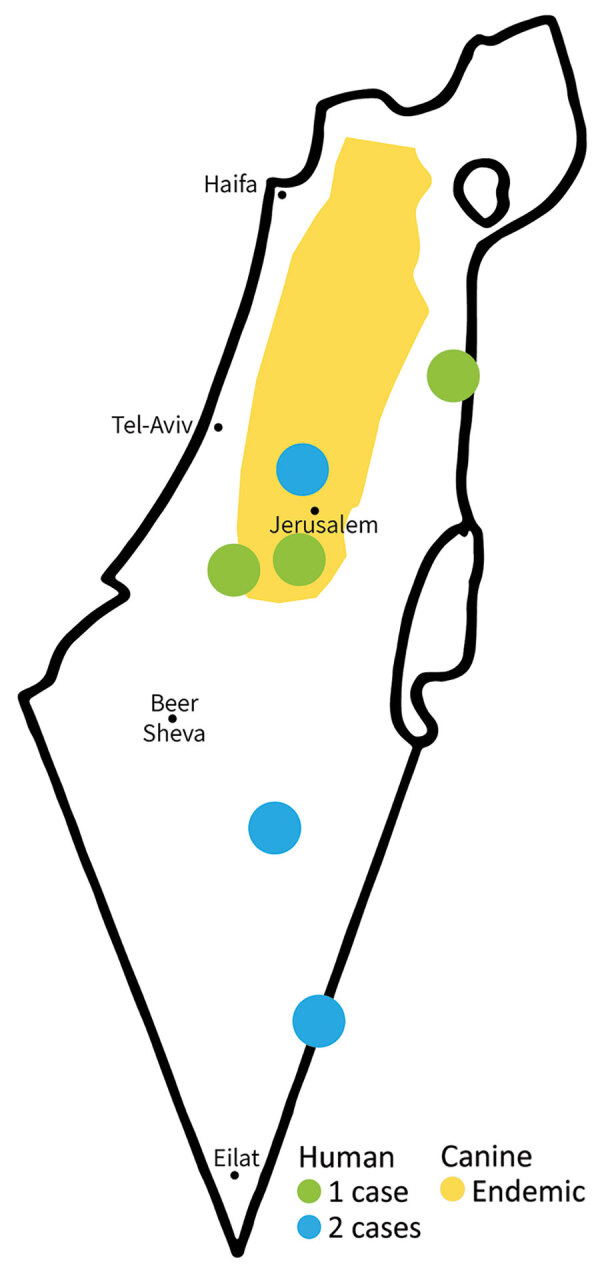
Foci of cutaneous leishmaniasis caused by *Leishmania infantum*, Israel, 2018–2021. Distribution of canine *L. infantum* in Israel based on Jaffe et al (*8*).

One case was diagnosed in 2018 and the other 7 cases during 2020–2021. Of the 8 patients, 6 were male and 2 female. The infections were acquired in different regions of Israel, including the southern region (Negev and Arava areas), where *L. major* is endemic, and the central region (east of Tel Aviv), where *L. tropica* is endemic (Figure 1).

**Figure 2:**
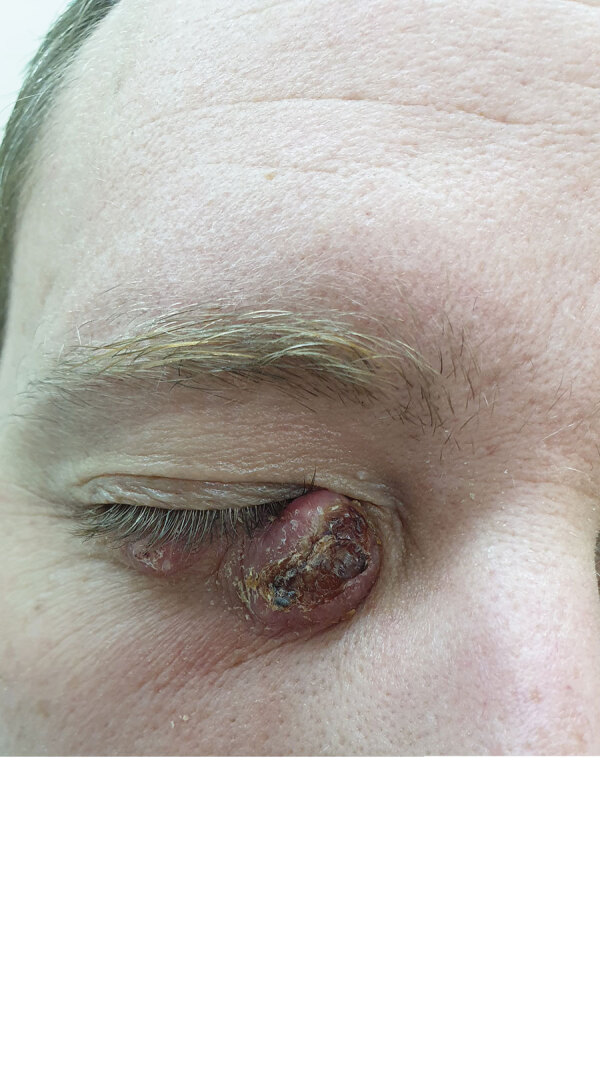
Cutaneous lesion caused by *Leishmania infantum* on the right lower eyelid of a patient seen at Chaim Sheba Medical Center, Israel, 2021.

The mean age of patients at diagnosis was 31 years (range 10–56 years; median 28.5 years). The average number of lesions was 2 (range 1–6). The lesions were commonly located on the limbs (6 patients); in 2 patients, the lesions were located on the face (Table 2; Figure 2).

**Table 2:** Clinical and demographic characteristics of 8 patients with cutaneous leishmaniasis caused by *Leishmania infantum*, Israel, 2018–2021

Characteristic	Value
Mean age, y (range)	31.8 (10–56)
Sex, no. patients	
M	6
F	2
Exposure area	Southern and eastern Israel
Mean no. lesions (range)	2 (1–6)
Anatomic location, no. patients*	
Head and neck	2
Upper limbs	3
Lower limbs	4

*Includes multiple locations per patient.

Two patients recovered within a short period (≈3 months) without any treatment, 3 patients were treated with topical therapy (intralesional sodium stibogluconate and liquid nitrogen), and the other 3 patients were treated with systemic therapy (oral miltefosine and intravenous liposomal amphotericin B). Systemic therapy was initiated when topical treatment failed or intralesional sodium stibogluconate injection was not feasible because of the anatomic location of the lesions (e.g., on the face or eyelid). All patients who received systemic therapy had a good response. None of the patients had systemic disease.

## Conclusions

The first known case of CL attributable to *L. infantum* infection in southern Israel was reported in 2016 (*6*). We describe a case series of 8 patients with *L. infantum* CL in Israel. The disease was acquired in different parts of Israel, and all but 1 case occurred during 2020–2021, pointing to a possible emergence of a new species causing CL in Israel. Canids, including domestic dogs and wild canids, are reservoirs for *L. infantum*. Recently, cats were also reported as a reservoir (*5*).

The known vectors of *L. infantum* are *Phlebotomus syriacus*, *P. tobbi*, and *P. perfilliewi* flies, all of which exist in Israel (*9*) Because both the reservoir and the vector for *L. infantum* have existed in Israel for years, the reason for this outbreak of CL is not well understood. Of note, in several countries, including Saudi Arabia, Turkey, and Yemen, *L. infantum* as a causative agent for CL has already been described (*10*–*12*), whereas Israel historically has not documented this disease.

Diagnosis of this *Leishmania* species in skin lesions can be made mainly by PCR testing. In Israel, PCR tests have been available since 2003 (*7*). At Chaim Sheba Medical Center, molecular diagnosis has been available since 2016, and it was only recently that the emergence of *L. infantum* CL was observed, thus excluding a diagnostic bias as a cause for this phenomenon. In addition, a query of 3 other centers in Israel where molecular diagnosis is performed indicated that cases of *L. infantum* caused by CL were seen only as of 2020 (E. Schwartz, pers. comm., email, 2022 Nov 1).

Clinical pleomorphism is a major feature of *Leishmania* parasites, a phenomenon particularly well illustrated in the case of *L. infantum*. This species is endemic in countries around the Mediterranean Basin and is most commonly known to cause VL, which is fatal if untreated. Host factors play an important role in this pleomorphism (*13*). However, some reports suggest a possible contribution of parasite factors attributable to different subspecies. Among the different *L. infantum* zymodemes (groups of strains showing the same isoenzymatic profile), some are restricted to cutaneous cases, such as MON-1, MON-24, MON-29, and MON-33 (*14*). However, the major and ubiquitous MON-1 zymodeme, mostly associated with VL, was also found in CL cases (*14*).

Transmission of *L. infantum* infection is considered predominantly zoonotic, and domestic animals are the major reservoir. The disease spreads through expansion of the zoonotic or the anthroponotic cycles (*15*) to regions where local vectors (phlebotomine species) can contribute to *L. infantum* transmission.

No specific recommendations exist regarding treatment of CL caused by *L*. *infantum*. The treatment regimen we chose was based on disease severity. In mild cases, no treatment was given, and the lesions healed spontaneously and rapidly within 3 months. In moderate cases, topical treatment was used (intralesional sodium stibogluconate and liquid nitrogen). In severe cases or in cases that were refractory to local treatment, systemic treatment was given (oral miltefosine and intravenous liposomal amphotericin B), all with good responses. None of the patients had systemic involvement. The treatment protocols for severe lesions were similar to those used in *L. donovani* CL cases and in severe cases of *L. major* and *L. tropica* CL. Although *L. infantum* can cause VL, none of the patients had systemic disease. Therefore, a nonsystemic treatment (topical or intralesional) seems to be an adequate treatment in most cases. 

In conclusion, the increasing number of *L. infantum* CL cases since 2020, occurring in different parts of Israel, point to an emerging new leishmania species in Israel. Clinicians should include this pathogen in the differential diagnosis of patients with cutaneous lesions clinically compatible with leishmaniasis.
